# Development of the clinical reasoning competency scale for nurses

**DOI:** 10.1186/s12912-023-01244-6

**Published:** 2023-04-25

**Authors:** Juyeon Bae, JuHee Lee, Mona Choi, Yeonsoo Jang, Chang Gi Park, Young Joo Lee

**Affiliations:** 1 Research Institute for Biomedical & Health Science, Department of Nursing, Konkuk University Glocal Campus, Chungwon-daero 268, Chungju-si, Chungcheongbuk-do 27478 Korea; 2 Mo-Im Kim Nursing Research Institute, College of Nursing, Yonsei University, Yonsei-ro 50, Seodaemun-gu, Seoul, 03722 Korea; 3 College of Nursing, University of Illinois Chicago, 845 S. Damen Ave., MC 802, #612, Chicago, IL 60612-7350 USA; 4 College of Nursing, Daegu Catholic University, 33, Duryugongwon-ro, 17-gil, Nam-gu, Daegu 42472 Korea

**Keywords:** Clinical reasoning, Scale, Nursing education, Validity, Reliability, Psychometric evaluation, Competency

## Abstract

**Background:**

Clinical reasoning is emphasized as an important component of nursing education, since nurses’ lack of clinical reasoning leads to incorrect clinical decision-making. Therefore, a tool for measuring clinical reasoning competency needs to be developed.

**Methods:**

This methodological study was conducted to develop the Clinical Reasoning Competency Scale (CRCS) and examine its psychometric properties. The attributes and preliminary items of the CRCS were developed based on a systematic literature review and in-depth interviews. The validity and reliability of the scale were evaluated among nurses.

**Results:**

The exploratory factor analysis was conducted for the construct validation. The total explained variance of the CRCS was 52.62%. The CRCS consists of 8 items for plan setting, 11 items for intervention strategy regulation, and 3 items for self-instruction. The Cronbach’s α of the CRCS was 0.92. Criterion validity was verified with the Nurse Clinical Reasoning Competence (NCRC). The correlation between the total NCRC and CRCS scores was 0.78, all of which were significant correlations.

**Conclusion:**

The CRCS is expected to provide raw scientific and empirical data for various intervention programs to develop and improve nurses’ clinical reasoning competency.

## Background

Clinical reasoning is a cognitive process used to make clinical judgments; in this process, a patient’s history is investigated, a physical assessment is performed, and the results are interpreted to design a health care plan [1, 2]. Nurses acquire information to solve the patient’s problem and combine this information with their knowledge to guide decision-making in patient care [3, 4]. Clinical reasoning involves incorporating the patient’s context and the clinical situation into critical thinking [5, 6]. Metacognition, which enables students to use a multidimensional strategy to search and consider an expanded range of possibilities to solve the problem considering the context [7], is a core attribute of clinical reasoning [8, 9].

A lack of clinical reasoning competency in nurses leads to incorrect clinical decision-making, which affects patient safety [10]. In contrast, the clinical reasoning competency of nurses enhances patient recovery and improves the quality of care. For this reason, a tool for measuring clinical reasoning competency needs to be developed and used. There exist two widely used tools for assessing nurses’ clinical reasoning: the Health Science Reasoning Test (HSRT) and the Nurse Clinical Reasoning Competence (NCRC) tool. The HSRT, which was developed by Insight Assessment in the United States, contains 33 questions that are completed in 50 minutes. The NCRC was developed in Taiwan [11], and it includes 15 items derived based on the clinical reasoning model [4]. However, these tools do not have metacognition attributes that check and evaluate the cognitive process in problem-solving, which is a key element of clinical reasoning competency. Thus, these tools have limitations in evaluating the multidimensional aspects of nurses’ clinical reasoning.

Previous studies have been conducted to improve clinical reasoning competency using educational methods such as simulation education and problem-based learning [12, 13]. Those studies indirectly measured clinical reasoning competency based on critical thinking [14] and problem-solving [15], which is insufficient. In research on pedagogy, metacognition refers to the control of actions related to obtaining and using information to improve inferential problem-solving. Metacognition helps learners to monitor their reasoning processes and regularly reflect on the process of cognition. It has been reported that activating metacognition improves problem-solving [16]. Therefore, this study was conducted to develop and verify the validity and reliability of the Clinical Reasoning Competency Scale (CRCS) for nurses.

## Methods

### Research design

This methodological research was conducted to develop the CRCS and examine its psychometric properties. The research was performed according to the methodology suggested by DeVellis [17].

### Research procedure

Permission for this study was obtained from the Institutional Review Board (IRB No. 2019-1741-001). The CRCS attributes and preliminary items were developed based on a systematic literature review and in-depth interviews. Built on these attributes, items to measure nurses’ clinical reasoning competence based on self-regulated learning theory were derived. The content validity of the CRCS was verified using the Delphi technique, and the validity and reliability of the scale were evaluated among nurses. Through this process, we aimed to build a scale with high reliability and validity that could measure nurses’ clinical reasoning competency.

### Theoretical framework

Self-regulation theory has been used to explain the cultivation of clinical reasoning competency. The theoretical basis of self-regulated learning theory is formed by constructivist teaching methods, according to which learners use systematic learning and metacognitive strategies to develop a clear goal or motivation for learning [13, 14]. This theory suggests that clinical reasoning competency can be fostered by controlling one’s actions until a goal is reached [18]. The development of a measurement scale should be based on a theoretical model to help researchers understand what to measure. Otherwise, the scale’s validity may be decreased, and data may be misinterpreted [19, 20]. A structured tool applying a theory can have an affirmative effect on intervention-based studies [21]. Thus, self-regulation theory was adopted for the development of the CRCS.

### Phase 1: analysis of the attributes of clinical reasoning competency

A systematic literature review was performed to identify the attributes and items of the CRCS. First, a literature search was performed for research articles on clinical reasoning measurements. The PubMed, Embase, MEDLINE Complete, Cumulative Index for Nursing Allied Health Literature (CINAHL) with full text, and RISS databases were searched. The keywords in the literature search were nur* AND clinical reasoning AND measur* and non-MeSH terms. The inclusion criteria were studies with clinical reasoning measurements and studies conducted among nurses and nursing students. The literature search was limited to the English and Korean languages. We excluded the gray literature. After searching the corresponding reference lists and abstracts, the full articles were collected and reviewed and manual searches in core journals were performed. The initial database search retrieved 442 studies, of which 326 remained after removing duplicates. After title and abstract review, 294 results were excluded, and the remaining 32 studies were assessed for eligibility. Seven studies were excluded because they were not conducted among nurses and nursing students or were not written in English or Korean. Twenty-five studies that satisfied the selection standards were identified, from which 14 studies were excluded because they did not measure clinical reasoning. Therefore, a total of 11 studies were included in the systematic review. In addition, the literature on self-regulation theory was analyzed to derive to attributes of clinical reasoning competency.

To supplement the concepts identified from the literature review and to develop items for an evaluation tool that would be suitable for the real-world circumstances faced by nurses, online in-depth interviews were conducted with 10 nurses. The CRCS is targeted at the estimate nurses’ clinical reasoning competency. Therefore, the in-depth interview was performed only by nurses. The nursing experts’ opinions were reflected in content validity. The inclusion criteria for the interviews were (1) nurses who understood the study purpose and (2) nurses who worked in hospitals with 500 beds or more. In-depth interviews were conducted with nurses with a range of experience, from less than 1 year of clinical experience to proficiency in the clinical career stage, as suggested by Benner [3]. The nurses worked in medical and surgical wards, the emergency room, and the intensive care unit. The in-depth interviews were guided using questions, such as “What is the difference in your nursing care, i.e., similarity or differences from nurses with high clinical reasoning competency?” and “Do you check the problem-solving process for a given patient and compensate for any deficiencies?” The interviews were conducted in a non-face-to-face format using Webex to protect the participants from the risk of coronavirus disease 2019 transmission. The interview data were analyzed using thematic analysis. The literature review and in-depth interview results are complementary. After each interview, the researchers recorded the critical points in writing. If there were no information to confirm, the decision was made not to perform more interviews considered as saturation.

### Phase 2: development of the item pool

Based on the results of the literature review and in-depth interviews, the attributes of clinical reasoning and preliminary items were derived. The CRCS was developed in the Korean language, and an expert on Korean language and literature revised it for readability and ambiguity. A 5-point Likert scale (1 = strongly disagree; 5 = strongly agree) was used as the response measure for the preliminary items.

### Phase 3: expert review with the delphi technique

To reach consensus on the derived items, the Delphi technique was used to collect expert opinions and reach an agreement. A group of 12 experts was formed that consisted of nursing professors, clinical professionals, and pedagogical experts. The pedagogical experts were involved in the psychometric evaluation of the instrument. Snowball sampling was done for the recruitment of Delphi experts. A second-round questionnaire was performed to examine the 72 preliminary items. The content validity ratio (CVR) was calculated based on the appropriateness reported by the expert panelists. The CVR is a measure of panelist agreement based on the proportion of panelists who rate an item as essential [22]. The CVR is calculated as follows:

$$CVR = [{n_e} - (N/2)]/(N/2)$$

In this equation, n_e_ is the number of the panelists who rate an item as essential, and N is the number of total panelists. Since there were 12 Delphi survey panelists, the minimum value was 0.56 [22]. Questions with a CVR of 0.56 or less were deleted.

### Phase 4: pilot study

After the completion of the preliminary CRCS, a pilot questionnaire was administered to 21 nurses to determine whether the instructions or any items were difficult to understand. The scale consisted of a 5-point Likert scale (1 = strongly disagree; 5 = strongly agree). The higher score indicates higher clinical reasoning competency.

### Phase 5: construct validity

Construct validity was examined using exploratory factor analysis (EFA). The participants of this study were nurses working in hospitals with more than 500 beds. The sample size is recommended to be 5–10 times the minimum number of items for factor analysis or an analysis of the correlations between items [23]. Considering the dropout, 500 nurses were recruited to participate in this study. Seventeen nurses did not complete the survey. Therefore, 483 nurses were included in the final EFA.

### Phase 6: criterion validity

Criterion validity was examined in terms of concurrent validity by comparing the CRCS with a “gold-standard” i.e., NCRC [11] clinical reasoning competency measurement [17]. The Cronbach’s α of the NCRC was 0.94, and the Korean version of the NCRC had a Cronbach’s α of 0.93 [24].

### Phase 6: reliability

Internal consistency was examined using Cronbach’s α. A test-retest assessment was conducted to verify its reliability. The test-retest was performed at 2-week intervals with the same subjects participating as in the construct validity survey. And the subjects who agreed with the participation, completed the in the retest study (n = 51).

### Data collection

Data collection was conducted through an online Google Survey of nurses working in hospitals with more than 500 beds. The researchers explained the study purpose, the inclusion and exclusion criteria, and the expected effects via communications distributed in the S University Hospital and nurse communities. In this study, convenience sampling was done to recruit nurses.

### Statistical analysis

The collected data were analyzed using SPSS for Windows version 25.0 (IBM Corp., Armonk, NY, USA). Descriptive statistics were used to analyze the participants’ demographic characteristics. We performed validity and reliability tests, and item analysis was conducted. Construct validity was determined using EFA. Principal component analysis and the varimax rotation method were used. Criterion validity was examined using Pearson correlation coefficients. Cronbach’s α was used to test the reliability. Test-retest reliability was assessed using Pearson correlation coefficients. Two researchers performed the data analysis.

## Results

### Identification of clinical reasoning competency attributes

According to the literature review, the components of self-regulated learning are metacognition, behavior, and environmental regulation. Metacognition, an essential element of clinical reasoning competency, consists of the properties of planning, monitoring, regulation, and evaluation [25, 26].

Behavioral regulation means that students choose meaningful behaviors by controlling their behaviors to reach goals. They achieve their goals through time management and self-examination activities, such as seeking help from others [27, 28]. Therefore, behavior regulation involves three factors: self-instruction to check the cognitive processes for problem-solving, self-reinforcement to enhance behavior, and help-seeking to obtain advice from colleagues. Three detailed factors were derived.

Environmental regulation refers to learners’ ability to sufficiently use materials such as books and the internet for effective learning in the problem-solving process. Kuiper [18] reported that relationships with other healthcare professionals include emphasizing the relationship with patients to facilitate multidisciplinary collaboration. Therefore, in the CRCS, empathy with the patient and multidisciplinary collaboration were derived as factors of environmental regulation to measure clinical reasoning competency.

These themes were derived by analyzing and organizing the results of in-depth interviews conducted to develop items to measure clinical reasoning competency (Table 1).

**Table 1:** Summary of the in-depth interviews

Attribution	Condensed meaning
Planning	Developing a plan for what to focus on before meeting the patient
Monitor	Evaluating patients in detail and organizing data
	Efforts to solve problems in an evidence-based nursing
Regulation	Finding problems by considering the patient’s changed situation
	Thoroughly reviewing the patient’s assessment and related data
Evaluation	Reflecting on the intervention provided to the patient
	Recording of reflections on patient care
Self-reinforcement	Continuous exploration for knowledge related to solving problems
	Sharing knowledge with colleagues
Self-instruction	Taking the time to record and organize what you don’t know
Help-seeking	Finding an expert who can help you and give advice
Empathy with patient	Providing sympathy and interest to the patient
	Listening to the patient’s discomfort
Multidisciplinary collaboration	Solving patients’ problems through collaboration with healthcare personnel

Through the literature review and in-depth interviews, three domains and nine clinical reasoning attributes were derived. The three domains were metacognition, behavioral regulation, and environmental regulation. The nine attributes were as follows: planning, monitoring, control, evaluation, self-reinforcement, self-instruction, help-seeking, empathy with the patient, and an interdisciplinary approach.

### Development of the preliminary item

In total, 72 preliminary items were developed based on the literature review and in-depth interviews. After two rounds of verification with the Delphi technique, 31 items were deleted because they had a CVR lower than 0.56 or were duplicates. The terminology was revised according to the experts’ opinions. For example, the item “analyze the reason for an error that occurred during nursing care” was changed to “analyze the cause of an error that occurred during nursing care.” The experts suggested phrasing this in terms of “cause” rather than “reason” in the questionnaire to estimate clinical reasoning competency, focusing on the process of metacognition. In total, 41 preliminary items were derived for the CRCS.

### Pilot study

The preliminary CRCS was administered as a questionnaire to 21 nurses. The participants suggested including the subject pronoun “I” to clarify the meaning of item #40. This item was modified. Finally, 41 preliminary CRCS items were confirmed.

### General characteristics of the participants

The validity and reliability of the CRCS were examined with 483 nurses A total of 91.5% of the participants were female, and the average age was 31.43 years old. The most common highest education level was a bachelor’s degree, the most common work department was the intensive care unit, and the most common range of clinical experience was between 5 and 10 years (Table 2).

**Table 2:** Characteristics of the participants (N = 483)

Characteristics	Categories	n(%)	Mean ± SD
Age			31.43 ± 5.52
Gender	Male	41 (8.5)	
	Female	442 (91.5)	
Marital	Unmarried	325 (67.3)	
status	Married	158 (32.7)	
Education	Diploma	31 (6.4)	
	Bachelor’s degree	358 (74.1)	
	Master’s coursework	35 (7.3)	
	Master’s degree or higher	59 (12.2)	
Work	Medical ward	132 (27.3)	
department	Surgical ward	82 (17.0)	
	Emergency room	20 (4.1)	
	Intensive care unit	167 (34.6)	
	Others	82 (17.0)	
Career	Less than 1 year	17 (3.5)	
(yrs)	1 to 3 years	93 (19.2)	
	3 to 5 years	125 (25.9)	
	5 to 10 years	137 (28.4)	
	More than 10 years	111 (23.0)	
Work	Shift work	392 (81.2)	
type	Non-shift work	91 (18.8)	

### Validity

Item analysis was performed using the mean, standard deviation, and item-total correlation coefficient. The appropriate range for the item-total correlation is 0.30–0.70 [29]. The item-total correlations of the CRCS questions ranged from 0.440 to 0.624.

To determine the number of factors of the 41 items, principal component analysis with varimax rotation was performed. The number of factors was determined based on the eigenvalues, scree plots, and parallel analysis results. In the parallel analysis, the three eigenvalues analyzed from the data were greater than those from randomly generated data, and three factors were extracted with this method. Accordingly, EFA was performed by selecting three factors, which explained 52.62% of the total variance. When the commonality was less than 0.40 or the factor loading value of two or more factors was 0.40 or more, the items were deleted for having common loadings; ultimately, 3 factors and 22 items were extracted (Table 3). The items included in the final three factors were reviewed, and the factors were named “plan setting,” “intervention strategy regulation,” and “self-instruction.”

**Table 3:** Explanatory factor analysis of preliminary items (N = 483)

Item	Factor 1	Factor 2	Factor 3	Communality
23. Identify the other method other to solve the patient's problem.	0.695	0.207	0.247	0.587
24. Reflect the anything wrong with the plan before intervention.	0.693	0.160	0.218	0.553
26. Repeatedly reflect the process of solving the patient's problem.	0.669	0.256	0.182	0.547
18. A different perspective on patient's health problems.	0.662	0.293	-0.058	0.528
27. Identify a better way to solve the problem even after solving the patient's problem.	0.649	0.096	0.307	0.524
25. Compare the results of the patient's problem solving with the target level.	0.645	0.169	0.071	0.449
21. Provide integrative interventions considering the patient and situation (e.g., family and environment).	0.635	0.190	0.131	0.456
22. Evaluate the nursing intervention.	0.596	0.295	0.212	0.487
10. A process of sufficient deliberation for interventions.	0.575	0.281	0.140	0.429
20. Collect additional data to close the gap between the information.	0.519	0.292	0.333	0.466
14. Continuously check the missing parts in solving the patient's problem.	0.512	0.352	0.126	0.402
6. Relate the knowledge to the information.	0.235	0.740	0.045	0.604
4. Prioritize problem solving strategies.	0.102	0.714	0.185	0.555
2. Discover important problems based on the data.	0.226	0.708	0.193	0.589
1. Distinguish the importance of the data.	0.189	0.687	0.270	0.580
5. Comprehensively grasp the relationship between the patient data.	0.279	0.683	0.145	0.566
9. Understand the overall patient situation.	0.233	0.632	0.139	0.473
12. Analyze the cause of an error during nursing care.	0.291	0.595	0.146	0.460
17. Find any problems in the care and correct them immediately.	0.281	0.578	0.056	0.416
33. Look for answers to questions don't know on my own.	0.199	0.250	0.741	0.654
35. Invest extra time to encounter problems to acquire don't know about the field of work.	0.299	0.168	0.733	0.654
32. Interested in acquiring new information related to the field of work.	0.178	0.188	0.730	0.600
Eigenvalue(total)	4.915	4.377	2.284	
% of variance	22.341	19.898	10.383	
Cumulative %	22.341	42.238	52.621	

Criterion validity was verified with the NCRC, which was developed by Liou et al. as a clinical reasoning scale [11]. The correlation between the total NCRC and CRCS scores was 0.78, and the correlations for the three CRCS factors were 0.76 for plan setting, 0.67 for intervention strategy regulation, and 0.50 for self-instruction, all of which were significant correlations.

### Reliability

The Cronbach’s α of the CRCS was 0.92, with the coefficients of the subdomains ranging from 0.73 to 0.89 (Table 4). The test-retest correlation coefficient was r = .76 (p < .001), indicating high reliability. After the completion of the above steps, the final CRCS consisted of 22 items of 3 factors. The factors of CRCS were plan setting, intervention strategy regulation, and self-instruction.

**Table 4:** The reliability of CRCS

Factor	Number of items	Range of score	Mean	SD	Cronbach’s α
Plan setting	8	15~40	31.67	4.02	0.87
Intervention strategy regulation	11	22~55	40.69	5.75	0.89
Self-instruction	3	3~15	11.26	1.95	0.73
Total	22	48~110	83.62	10.15	0.92

Abbreviations: CRCS, Clinical Reasoning Competency Scale

## Discussion

This study was conducted to develop a scale to measure nurses’ clinical reasoning competency. The validity of the scale was verified. The final CRCS was confirmed through reliability testing.

This scale was developed based on self-regulation learning theory, which suggests that nurses can cultivate clinical reasoning competency by controlling their actions until they reach their goals or foster a motivation for problem-solving. Self-regulation learning consists of metacognition, behavior regulation, and environmental regulation [18]. These three attributes proposed by self-regulation learning theory do not work independently; instead, their interaction allows problem-solving to be reliably achieved [30]. The previous tools to measure the clinical reasoning competency of nurses or health care practitioners mostly focused on knowledge to solve problems or cognitively oriented questions about making an accurate diagnosis. Thus, these tools do not include the metacognitive aspects of clinical reasoning such as reflecting upon and regulating the problem-solving process. In nurses’ clinical decision-making through reasoning, metacognition refers to the ability to set cognitive strategies, knowing which outcomes are produced. Thus, metacognition is a key element for problem-solving [31, 32]. Metacognition, which can enable nurses to make immediate judgments about their problem-solving through reflection and evaluation, is an essential predictor of clinical reasoning competency. Therefore, the CRCS is valuable because it includes the attribute of nurses’ metacognitive competency based on self-regulated learning theory. In addition, clinical reasoning competency should be measured based on decision-making or observable behavior. The CRCS, which was developed as a structured scale by applying the theory of self-regulated learning, is expected to have a positive impact on conceptual models and intervention strategies to enhance researchers’ understanding of clinical reasoning competency. In this study, based on a literature review and in-depth interviews, 72 preliminary questions were derived: 44 metacognitive questions, 22 behavioral regulation questions, and 6 environmental regulation questions. Item analysis was conducted to verify the means, standard deviations, and correlation coefficients between the questions and the entire CRCS [29].

As a parallel analysis, EFA was performed using varimax analysis, resulting in 22 items with a three-factor structure 52.62% of the total explained variance of the three factors, i.e., plan setting, intervention strategy regulation, and self-instruction. Plan setting was suggested as the first factor. This factor consists of 8 items. It was named plan setting. Most of the items are related to plans to provide nursing care for the patient. Lee et al. [12] analyzed the techniques and processes of clinical reasoning among nurse practitioners. Nurses identified patients’ health problems quickly in complex clinical practice situations and established solutions and plans for cognitive strategies. This is a fundamental step in clinical reasoning. The clinical reasoning competency of nurses is imperative for the step of setting a plan as part of the overall cognitive strategy for problem-solving. The CRCS can estimate this attribute.

The second factor, intervention strategy regulation, had the highest explained variance. The intervention strategy regulation factor was extracted by integrating evaluation and control attribution from the preliminary CRCS. Among the 11 items, six are related to control attribution. During problem-solving in a difficult situation, seeking advice from the nurse’s colleagues or modifying one’s cognitive strategies are emphasized as essential [33]. It was named factor as intervention strategy control. Therefore, the items included in intervention strategy regulation are considered to be meaningful for measuring nurses’ clinical reasoning competency.

The third factor was self-instruction. This factor includes 3 items. It was named self-instruction. This factor consists of items measuring the self-development of nurses for developing clinical reasoning competency. In self-regulated learning, Paul and Pintrich [34] reported that learners need to observe themselves to achieve their own goals. Using self-regulated learning strategies, nurses discovered problems, corrected them, and developed behavioral control [8]. The CRCS makes it possible to measure nurses’ strategies for problem-solving.

In order to identify clinical reasoning competency factors, attributes were derived through a literature review, and the CRCS of nurses was composed of three factors as a result of the Delphi technique and the tool verification process. The deleted factor as help-seeking attribution as a component of behavioral regulation was integrated with the multidisciplinary collaboration component of environmental regulation. It is considered that the concept of seeking advice from and collaborating with other colleagues overlaps with seeking help in the process of problem-solving. The monitoring attribute was incorporated into plan-setting, and assessments were integrated into interventional strategy regulation. Participants may have reviewed the appropriateness of cognitive strategies as part of the process of establishing methods for problem-solving. Furthermore, nurses reflected on errors and modified their cognitive strategies when solving patients’ health problems, which could have contributed to the integration of the factors of the evaluation item with the factors of intervention strategy regulation.

To verify criterion validity, we used the NCRC developed by Liou et al. [11] based on a clinical reasoning model [4]. The correlation coefficients between the two scales were r = 0.52–0.78, indicating that the criterion validity was satisfactory.

The reliability test of the CRCS demonstrated a Cronbach’s α = 0.92. For each factor, the Cronbach’s α was > 0.70, indicating satisfactory reliability following the criterion proposed by Nunnally and Bernstein [35]. According to this criterion, the Cronbach’s α of the CRCS was satisfactory.

The CRCS was developed to measure the clinical reasoning competency of nurses. A higher score indicates higher clinical reasoning competency. The CRCS is a standardized tool that was verified through the Delphi method and showed satisfactory construct validity, criterion validity, and reliability. Therefore, the CRCS can be used in various studies to develop and cultivate nurses’ clinical reasoning competency.

### Limitations

The scale developed in this study was used to collect primary data that can improve clinical reasoning competency. Based on the research results, the limitations and suggestions of this study are as follows.

First, there may have been restrictions in the selection or comparison of nurses with clinical reasoning competency above a certain level. A cutoff value of the CRCS was not presented. We suggest supplementing the subjective judgments of respondents and determining the sensitivity and specificity of the scale to increase its usefulness in follow-up studies.

Second, the difficulty of the items was not analyzed. Therefore, further research is needed to supplement the tool through an elaboration process.

Third, since this study used convenience sampling of nurses working in general hospitals with more than 500 beds, there may have been limitations related to bias or a lack of generalizability of the data. To use the CRCS among nurses working in other environments, the validity and reliability of the scale will need to be further verified.

## Conclusion

This methodological study was conducted to develop a scale to measure nurses’ clinical reasoning competency. Preliminary questions were derived by analyzing the attributes of nurse’s clinical reasoning competency with a literature review and in-depth interviews. The validity of the scale was verified using the Delphi technique. The preliminary CRCS was completed through a pilot study. The final CRCS was completed by verifying the construct validity, criterion validity, reliability, and test-retest reliability of the scale with various methods based on data from 483 nurses working in hospitals with 500 beds or more. The CRCS is a self-reported measurement tool consisting of 11 items for intervention strategy regulation, 8 items for plan setting, and 3 items for self-instruction. All items are scored on a 5-point Likert scale, with higher scores indicating higher clinical reasoning competency. The average CRCS score was 83.62 in this study. The average score for each factor was 40.69 for intervention strategy regulation, 31.67 for plan setting and 11.26 for self-instruction. The CRCS was shown to have validity and reliability in measuring nurses’ clinical reasoning competency. Therefore, it is expected that using the CRCS will provide raw scientific and empirical data for various intervention programs to develop and improve nurses’ clinical reasoning competency. In addition, the attributes of clinical reasoning competency identified in the development of this scale can be applied to develop educational programs for nurses’ clinical reasoning competency and improve their problem-solving. Ultimately, this research is expected to contribute to patient safety.

## Data Availability

The authors can confirm that all relevant data are included in the article.

## List of abbreviations

CINAHL: Cumulative Index for Nursing Allied Health Literature
CRCS: Clinical Reasoning Competency Scale
CVR: content validity ratio
EFA: exploratory factor analysis
GFI: goodness-of-fit index
HSRT: Health Science Reasoning Test
NCRC: Nurse Clinical Reasoning Competence
